# Comparison of Ignition Process and Thermodynamic Conditions of TC4 and TC17 Alloys Under High-Speed Rubbing Ignition

**DOI:** 10.3390/ma18010016

**Published:** 2024-12-24

**Authors:** Yajun Li, Jianjun Li, Zichong Zu, Congzhen Wang, Yuqi Zhang, Lei Shao, Jinfeng Huang

**Affiliations:** 1 State Key Laboratory for Advanced Metals and Materials, University of Science and Technology Beijing, Beijing 100083, China; liyajunustb@163.com (Y.L.); lijjustb@163.com (J.L.); zuzichong@126.com (Z.Z.); wcz202412@163.com (C.W.); zhang18438698876@163.com (Y.Z.); 2 School of Materials Science and Engineering, Shandong University of Science and Technology, Qingdao 266590, China; shaoleiustb@163.com

**Keywords:** rubbing, titanium alloy, critical condition, combustion

## Abstract

This study investigates the combustion characteristics and critical thermodynamic conditions for the ignition of TC4 and TC17 alloys under high-speed friction conditions. The results indicate that, under identical rubbing conditions, both the critical pressure and the ignition temperature of the TC17 alloy are higher than those of the TC4 alloy. The critical ignition conditions for both alloys increase with thickness, while they decrease with increasing rotational speed, oxygen concentration, and oxygen pressure. The primary characteristics in the initial stage of friction ignition are abrasive and adhesive wear. As the duration of friction increases, material from the friction surface begins to peel away, creating favorable conditions for ignition. At the moment of ignition, significant peeling occurs, along with visible cracks and molten structures, resulting in the production of a substantial amount of titanium oxide on the friction surface. Based on the ignition theory proposed by Frank-Kamenetskii, the reaction order, adsorption coefficient, pre-exponential factor, and activation energy of the ignition criterion under high-speed friction conditions were determined by fitting and analyzing the experimental results. The ignition temperatures of the TC4 and TC17 alloys at different speeds were predicted with a relative error of less than 2.06%. This demonstrates that the Frank-Kamenetskii model can be utilized to explain the critical ignition conditions of titanium alloys under high-speed rubbing conditions.

## 1. Introduction

The improvement of aircraft performance cannot be separated from the development of advanced materials. Titanium alloy offers several advantages, including high specific strength, resistance to high temperatures, corrosion resistance, and excellent performance at low temperatures. It is an essential material in aerospace [1], biomedicine [2], and various other fields [3], and is widely utilized in components such as engine rotor blades, casings, turbine disks, and more [1]. Titanium alloy has good high-temperature properties under normal circumstances; however, there are various factors that can cause titanium or titanium alloy parts to ignite (such as severe impact and friction). In these events, damage or burning is caused. The spread speed of “titanium fire” is fast, taking only 5–20 s from the beginning to the end of combustion [4]; consequently, it is difficult to take firefighting measures [5]. Therefore, it is crucial to clarify the ignition conditions of titanium alloys under extreme conditions to ensure the safe use of these materials.

Littman et al. [4] investigated the phenomenon of spontaneous combustion in pure titanium under different oxygen concentrations; they determined the effect of temperature on the spontaneous combustion limit and revealed the relationship between the critical oxygen pressure and the temperature. It was found that metallic materials could only spontaneously ignite if they contained oxides that were soluble in the metal. Additionally, ignition occurred when the initial reaction was sufficiently intense to raise the surface temperature to the melting point or eutectic point of the metal. Shao et al. investigated the combustion behavior and kinetics of TC4 [6], TC11 [6], and Ti14 alloys [7] using non-isothermal oxidation tests, promoted ignition combustion (PIC) tests, and intermittent combustion tests. The enrichment and distribution of the alloying elements near the solid–liquid interface significantly affected the burning rate of the titanium alloys [8]. The formation of titanium oxide (TiO) through the peritectic reaction between the liquid phase and the alpha (α) phase was a key factor in the enhancement of the burning rate [9]. Bolobov et al. proposed a mechanism to explain the auto-ignition of titanium alloys with the formation of a juvenile surface of the metal in oxygen at elevated pressure [10]. Upon structural failure, the auto-ignition of titanium alloys in oxygen was caused by the self-heating of the failure fragments with a juvenile surface to the melting point of the material due to the heat of adsorption and the dissolution of oxygen in the metal [11]. The total rate of the interaction was determined according to the rate of oxygen adsorption on the juvenile surface and depended on the oxygen pressure [12]. Khaikin et al. [13] found that when the oxide film on titanium particles ruptured and lost its protective properties, the particles could ignite. Based on the Semenov model, a relationship was established between the thickness of the titanium oxide layer and the ignition temperature [14]. The calculation of heat distribution during solid heating primarily involves the Fourier heat transfer model [15] and heat distribution based on fractional order theory [16]. Given the complexity of heat distribution under the thermo-mechanical coupling of rubbing conditions, the Fourier heat transfer model is currently the main approach used for simulating and calculating the temperature field. Mi et al. [17] conducted research on titanium alloys through friction ignition methods. A theoretical thermal model for the friction heat source of titanium alloy was developed, and the corresponding equations for critical temperature and ignition delay time were derived [18]. Liang et al. [15] conducted model calculations on the ignition process of titanium alloys under three heating conditions: isothermal heating, non-isothermal linear heating, and non-isothermal friction heating. They examined the impact of various environmental factors, including initial heating temperature, heating rate, oxygen concentration, and flow rate, on ignition parameters. Utilizing heterogeneous ignition theory, they developed a titanium alloy ignition model that incorporates the friction heat source. They derived theoretical formulas for calculating ignition temperature and ignition delay time and further analyzed how factors such as friction coefficient, contact radius, and flame retardant layer influence ignition parameters [19].

Frank-Kamenetskii established an ignition model in which a temperature gradient existed within the sample, and the heat transfer process adhered to Fourier’s law [20]. Thomas [21] extended the Frank-Kamenetskii model to the case where there was a temperature gradient between the reactant temperature and the ambient temperature at the boundary, and the difference in the heat transfer at the boundary could be expressed and converted using the Biot number (Bi). Afterward, Gray et al. further improved the model in terms of reactant consumption and expanded the application scope of the Frank-Kamenetskii model [22]. This theory model has been used for the combustion of coal [23], compounds [23], batteries [24], etc. Wang et al. [25] compared and analyzed the applicable conditions of the Semenov and Frank-Kamenetskii theories on the promotion of ignition conditions. Based on the ignition model derived from the theories of Semenov and Frank-Kamenetskii, along with an analysis of the experimental data, it was found that the Frank-Kamenetskii model more accurately described the effects of size, oxygen concentration, and oxygen pressure on the ignition temperature and critical oxygen pressure of the TC17 alloy [26].

The research mentioned above significantly contributes to a deeper understanding of the ignition phenomenon in titanium. However, the combustion experiments and theoretical analyses previously conducted do not accurately represent the actual rubbing conditions of aero-engines, leading to a substantial discrepancy between the research and the real-world scenarios. Furthermore, there is currently no established theoretical framework that provides a satisfactory description of this phenomenon. Therefore, this study employs a high-speed rubbing experimental device to closely replicate the actual working conditions of titanium combustion, and it incorporates various factors that influence ignition under high-speed rubbing conditions—such as thickness, oxygen pressure, oxygen concentration, and rotational speed—into a mathematical model based on Frank-Kamenetskii theory. The advantages of TC4 alloy include low density and good strength and fatigue performance, as well as a simple composition. It is suitable for use at temperatures ranging from 300 to 350 °C and is primarily used in the manufacturing of key components such as blades, airframe structural parts, and landing gear for aircraft engines. In contrast, TC17 alloy offers high strength, good hardenability, and excellent fracture toughness. Its long-term service temperature can reach up to 427 °C, making it ideal for applications such as low-pressure compressor disks and fans in aero-engines. This study determines the parameters of the ignition criterion within the model and quantitatively describes the effects of various factors on the critical ignition condition of TC4 and TC17 alloys under high-speed rubbing conditions. Based on this theoretical model, the ignition temperatures of the TC4 and TC17 alloys at different rotational speeds are predicted, thereby validating the effectiveness of the ignition criterion.

## 2. Experimental Details

### Experimental Material

In this work, the typical titanium alloy TC4 and TC17 alloys were supplied by the Central Iron and Steel Research Institute. The chemical compositions of the TC4 and TC17 alloys are shown in Table 1. The TC4 and TC17 alloys used in this article are commercial alloys designed for aero-engine applications. Their mechanical properties at both room and elevated temperatures meet the national standard [27] (GJB2218A-2018), as shown in Table 2. A series of flake specimens were cut from the ingot using Wire Electrical Discharge Machining (WEDM). The specimens measured 30 mm in length and 8 mm in width, with a thickness ranging from 2 mm to 8 mm. The surface of each rubbing specimen was polished with 2000# sandpaper and ultrasonically cleaned in alcohol for approximately 10 min.

**Figure 1:**
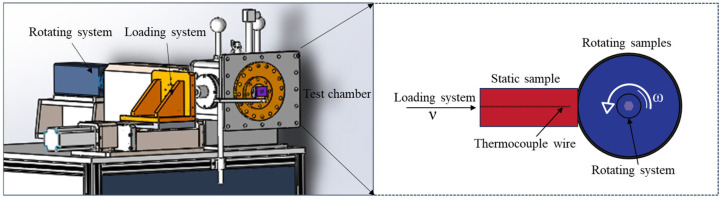
3D representation and schematic diagram of equipment.

The high-speed rubbing test device, model MTZ-1, was designed by our team and manufactured by Zhangjiakou Chengxin Equipment Co., Ltd. in Zhangjiakou, China. It features a maximum rotational speed of 3000 rpm, a maximum load capacity of 500 N, and can withstand a maximum gas pressure of 3 MPa. The schematic diagram of the high-speed rubbing device is shown in Figure 1. The rotor system is connected to a high-speed motor via a transmission shaft, enabling rapid rotation of the rotor disc during the high-speed rubbing test. The rotor disc is constructed from TC4 titanium alloy and has a diameter of 100 mm and a thickness of 8 mm. The feeding system propels the stator sample toward the rotor disc, resulting in rubbing and applying radial pressure (i.e., load) during the process. When designing the friction test device, we analyzed the actual working conditions of the aircraft engine during a titanium fire. This analysis was combined with the technical parameters of the testing equipment to select the following testing parameters: a speed range of 500 to 2000 rpm, a load range of 50 to 100 N, and a duration of 3 s. The entire ignition process was recorded by a high-speed camera (pco.dimax S4, Excelitas, Kelheim, Germany) at a capture frequency of 1000 frames per second. Under specific oxygen pressures, titanium alloys can ignite. If an alloy fails to ignite after five consecutive attempts at a given oxygen pressure, it is deemed that the alloy will not combust at that pressure. Consequently, there is a minimum oxygen pressure at which titanium alloys do not ignite, referred to as the critical oxygen pressure for titanium alloy combustion. The critical ignition pressure is measured using a specialized high-sensitivity gas temperature and pressure sensor. This gas pressure sensor has a measurement range of 0 to 10 MPa and an accuracy of ±0.1%. According to a previous study, there is a sudden change in temperature during the ignition process of titanium alloys [28]. It is believed that the temperature at the moment of this sudden change represents the critical ignition temperature. Here, the critical ignition temperature is measured using the MCS640 high-speed infrared camera from Luma Sense Technology, Denver, CO, USA. This camera has a temperature measurement range of 600 °C to 3000 °C, a capture frequency of 60 frames per second, and a measurement accuracy of ±0.5%. The load during the rubbing process of the titanium alloy is measured using a customized two-dimensional piezoelectric force sensor. This sensor has a measurement range of 0 to 10 KN in both the X and Y directions, with a measurement accuracy of ±0.1%. The displacement during the rubbing process of the titanium alloy is measured using a customized eddy current displacement sensor. This sensor has a measurement range of 0 to 2 mm and an accuracy of ±0.1%. The concentrations of oxygen, nitrogen, and argon used in the experiment were all 99.9%.

## 3. Results

### 3.1. High-Speed Rubbing Ignition Process and Combustion Behavior

**Figure 2:**
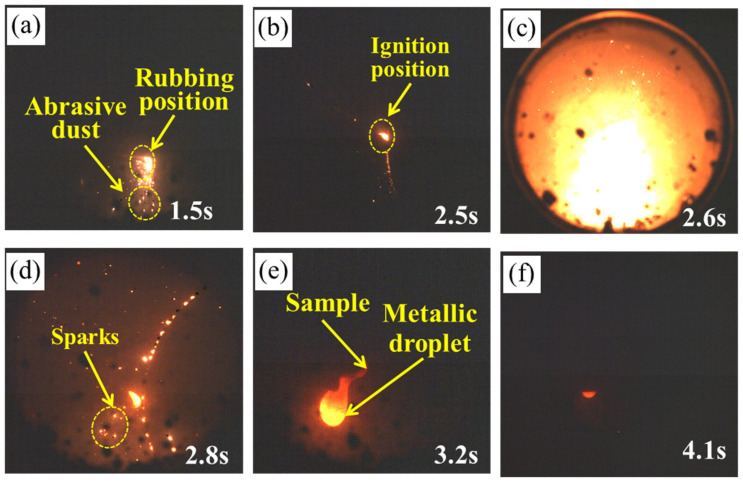
The combustion process of TC4 alloy samples: (**a**–**c**) ignition stage; (**d**,**e**) intense combustion stage; (**f**) combustion extinction phase.

**Figure 3:**
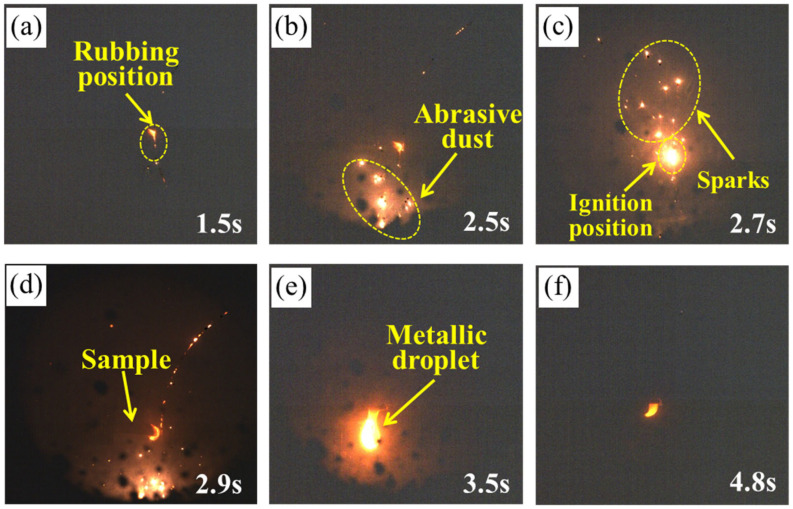
The combustion process of TC17 alloy samples: (**a**–**c**) ignition stage; (**d**,**e**) intense combustion stage; (**f**) combustion extinction phase.

The rubbing ignition process of the TC4 and TC17 alloys recorded using a high-speed camera is shown in Figure 2 and Figure 3, respectively. As shown in Figure 2a, a significant amount of debris is generated once the TC4 alloy begins to rub. This debris is expelled from the rubbing surface and subsequently ignites. Heat accumulation at the edge of the rubbing surface occurs rapidly, leading to the initial ignition, which spreads throughout the entire specimen, as illustrated in Figure 2b. Figure 2c illustrates the TC4 alloy at the moment of ignition caused by rubbing. It can be observed that when the TC4 sample begins to ignite at 2.6 s, a bright white light is emitted, accompanied by the sputtering of droplets. Figure 2d–f show the combustion, expansion, and extinguishment processes of the specimen. It is evident that during the expansion stage, a molten pool develops at the end of the specimen, accompanied by the splattering of sparks. As combustion progresses, molten metal droplets start to fall, and this cycle continues until the specimen is entirely consumed. The time from the formation of the molten pool to the first droplet dripping is about 0.4 s, as shown in Figure 2d,e. As shown in Figure 3a, from 1.5 s, the temperature at the edge of the friction surface increases, and the amount of debris is less than that seen with the TC4 alloy. After 2.5 s of rubbing, the amount of debris increases, with debris being expelled for combustion, as shown in Figure 3b. When the local temperature in the contact area exceeds the ignition point of the TC17 titanium alloy, the sample ignites, as shown in Figure 3c. At this point, it emits a brilliant white light and generates numerous sparks that radiate outward from the burning sample. In comparison to TC4, the brightness and size of the sparks are smaller. After ignition, the time from the formation of the molten pool to the first drop of molten droplet is approximately 0.6 s, which is longer than that of TC4. By comparing the combustion phenomena of the two alloys, it was observed that TC4 exhibited more intense ignition and combustion expansion than TC17. This was evidenced by larger sparks at the moment of ignition, increased droplet splashes during the combustion expansion process, and a quicker formation of the molten pool and droplets.

**Figure 4:**
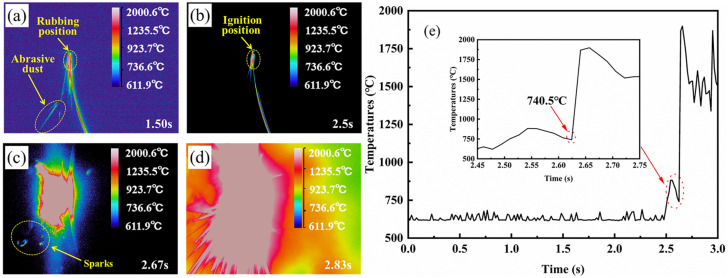
In situ thermal imaging of TC4 alloy during rubbing ignition process: (**a**–**d**) thermal imaging; (**e**) ignition temperature curve.

**Figure 5:**
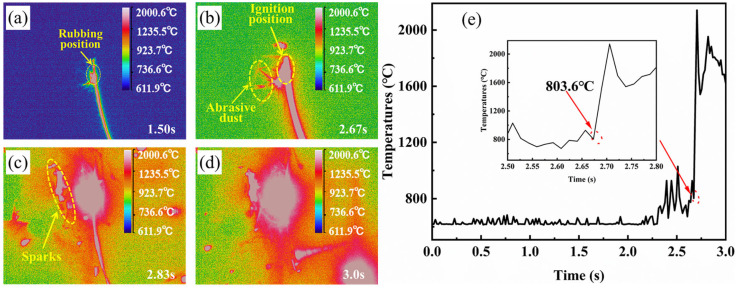
In situ thermal imaging of TC17 alloy during rubbing ignition process: (**a**–**d**) thermal imaging; (**e**) ignition temperature curve.

Figure 4 displays the thermal image of the TC4 alloy during the rubbing ignition process. From 1.5 s, it is evident that the temperature at the edge of the rubbing surface gradually increases, causing debris to be expelled and ignited, as illustrated in Figure 4a. The sample was not ignited at 2.5 s; however, it ignited at 2.67 s and began to expand. This indicates that the sample was ignited during this time period, as shown in Figure 4b,c. It can also be observed from the temperature change curve during the rubbing process that the ignition time of the sample is approximately 2.61 s. At this moment, the temperature rises sharply to about 2000 °C, as shown in Figure 4e. The temperature immediately preceding the sudden change point is referred to as the ignition temperature, which is 740.6 °C. As the combustion progresses, the combustion area of the specimen emits a brilliant white light, accompanied by the splattering of burning droplets, as depicted in Figure 4c. At 2.83 s, the thermal imaging display indicates that the sample continues to burn, heating the surrounding air, with the flame temperature exceeding 1800 °C at this moment. It can be observed that the TC17 sample is locally ignited at 2.67 s from Figure 5b, after which the ignition rapidly spreads throughout the entire sample (Figure 5c). The temperature variation curve during the friction process shows a sudden increase in temperature at this point, which is identified as the critical ignition temperature of approximately 803.6 °C (Figure 5e). Under the same test conditions, TC17 exhibits a higher ignition temperature than TC4. Following ignition, the thermal imaging reveals that the burning sample heats the surrounding air, with the maximum temperature reaching 2000 °C.

**Figure 6:**
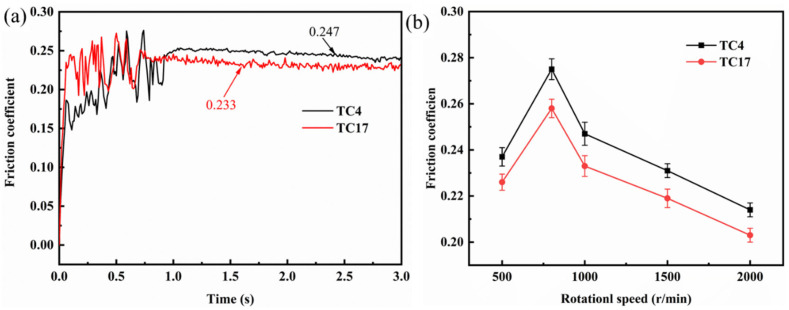
The friction coefficient during rubbing ignition process: (**a**) friction coefficient curve; (**b**) average friction coefficient.

Figure 6a depicts the friction coefficient curve observed during the rubbing process at a load of 200 N and a rotational speed of 1000 r/min. As can be seen from the figure, under identical test conditions, the friction coefficient of TC4 is higher than that of TC17. Both titanium alloys demonstrated considerable fluctuations in their friction coefficients at the onset of rubbing, indicating that they were in the pre-wear stage. As the rubbing continued, following a specific duration of pre-wear, the wear process transitioned into a stable stage, and the friction coefficient also tended to stabilize. According to the statistics on the friction coefficient during the stable phase, it was observed that the average friction coefficient of the two alloys initially increased and then decreased with an increase in rotational speed, reaching its maximum at 800 r/min, as illustrated in Figure 6b. The maximum friction coefficients for the TC4 and TC17 alloys were 0.275 and 0.258, respectively. This may be due to the transition of the wear mechanism from abrasive wear to adhesive wear as the speed increases from 500 r/min to 800 r/min. This transition causes adhesion on the rubbing surface, which in turn increases the friction coefficient. As the speed continues to rise, the wear mechanism shifts from adhesive wear to oxidative wear, resulting in the formation of a protective oxide layer on the rubbing surface [29], thereby reducing the friction coefficient [30].

### 3.2. Microstructure and Composition During Rubbing Ignition Process

**Figure 7:**
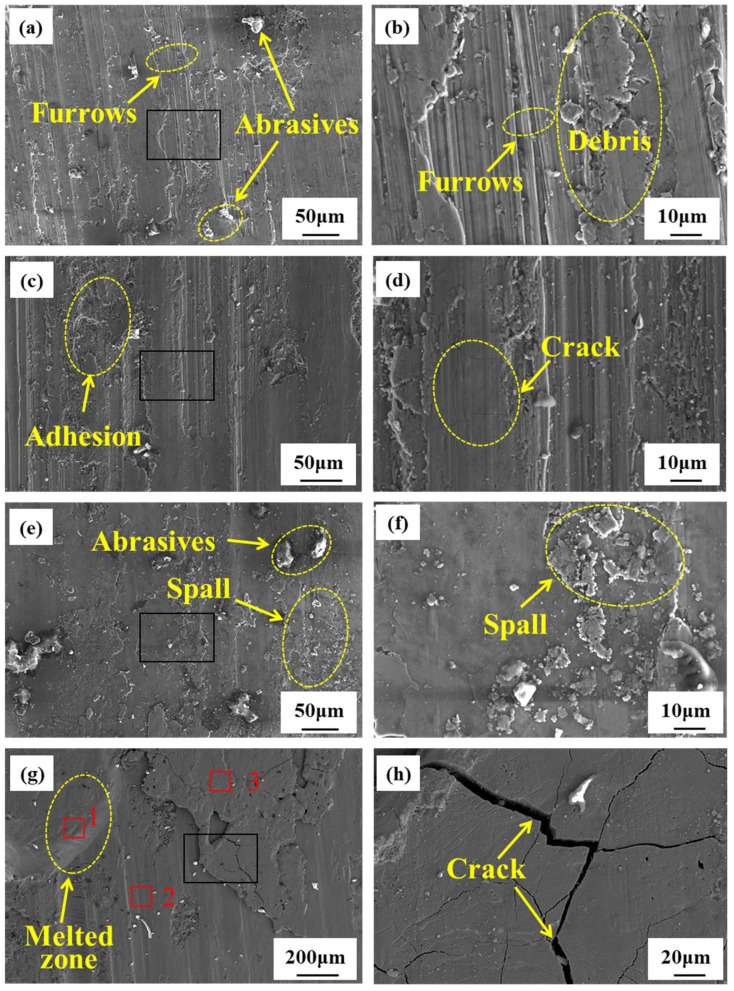
Microstructure of TC4 alloy during rubbing ignition process: (**a**,**b**) 0.5 s; (**c**,**d**) 1.5 s; (**e**,**f**) 0.5 s; (**g**,**h**) ignition moment.

**Figure 8:**
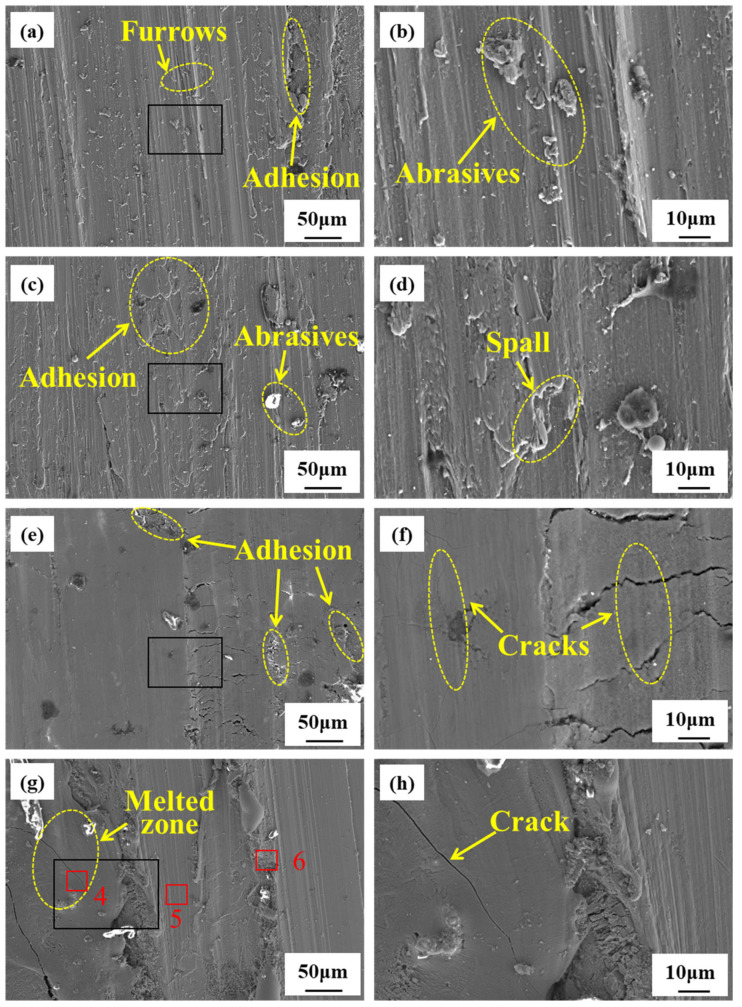
Microstructure of TC17 alloy during rubbing ignition process: (**a**,**b**) 0.5 s; (**c**,**d**) 1.5 s; (**e**,**f**) 2.5 s; (**g**,**h**) ignition moment.

The morphology of the rubbing surface during the friction ignition process of the TC4 and TC17 alloys is shown in Figure 7 and Figure 8. As can be seen in Figure 7a,b, at 0.5 s after the initiation of rubbing, the surface exhibits scratches, along with a significant number of white abrasive particles and plowed grooves, which primarily result from abrasive wear. As the rubbing continues, a substantial amount of debris adheres to the surface, and the metal on both sides of the plowed groove experiences plastic deformation and is displaced outward. As wear progresses, the displaced material is flattened once more, and this cycle of plastic deformation contributes to the formation of cracks (see Figure 7c,d). Figure 7e,f depict the microscopic morphology after 2.5 s of rubbing. As the temperature rises, the material softens, and deformation accumulates continuously, leading to the expansion of burn cracks and the detachment of thin flakes, which results in delamination wear. Figure 7g,h display the microscopic morphology at the moment of ignition. At this point, molten structures and wider cracks are visible on the rubbing surface. Combined with the compositional analysis presented in Table 3, it can be inferred that the rubbing surface contains a significant amount of oxides at this stage. As shown in Figure 8a,b, 0.5 s after the onset of rubbing, the rubbing surface displays numerous grooves and a small amount of adhesion, with wear particles that are smaller in size compared to those of TC4. At this stage, the wear mechanism is primarily abrasive wear. As the rubbing continues, the size of the wear particles increases, the adhesion traces become more pronounced and spalling begins to occur (see Figure 8c,d). Figure 8e,f present the micro-morphology of the TC11 alloy at 2.5 s after the onset of rubbing, revealing a relatively smooth rubbing surface with cracks extending to the surface, but no material spalling, which indicates oxidative wear. Figure 8g,h depict the micro-morphology of the TC11 alloy at the moment of ignition, where melting structures and cracks are also observable, along with the formation of a significant amount of TiO and TiO_2_.

**Figure 9:**
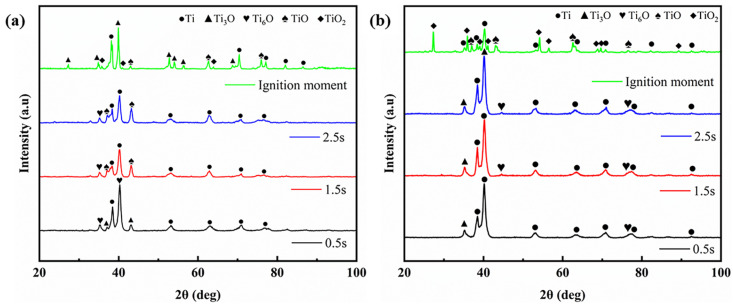
XRD analysis during rubbing ignition process: (**a**) TC4; (**b**) TC17.

The phase analysis of the two alloy rubbing surfaces during the rubbing process is shown in Figure 9. As can be seen in Figure 9a, at 0.5 s after the initiation of rubbing, the primary phases present on the TC4 alloy rubbing surface are Ti, Ti_3_O, and Ti_6_O. At 1.5 s, TiO and TiO_2_ begin to emerge. During ignition, the concentrations of TiO and TiO_2_ increase significantly at the moment of ignition; this corresponds to the rise in oxygen content on the rubbing surface, as indicated in Table 3, and the melting structure of the rubbing surface depicted in Figure 7g. Figure 9b presents the X-ray diffraction (XRD) analysis of the TC17 alloy rubbing surface during the ignition process. Prior to the ignition of rubbing at the three time points, there were no significant changes in the phases of the rubbing surface, which primarily consists of Ti, along with minor amounts of Ti_3_O and Ti_6_O. Only at the moment of ignition does the content of TiO and TiO_2_ increase markedly.

### 3.3. Critical Ignition Conditions of TC4 and TC17 Alloys

**Figure 10:**
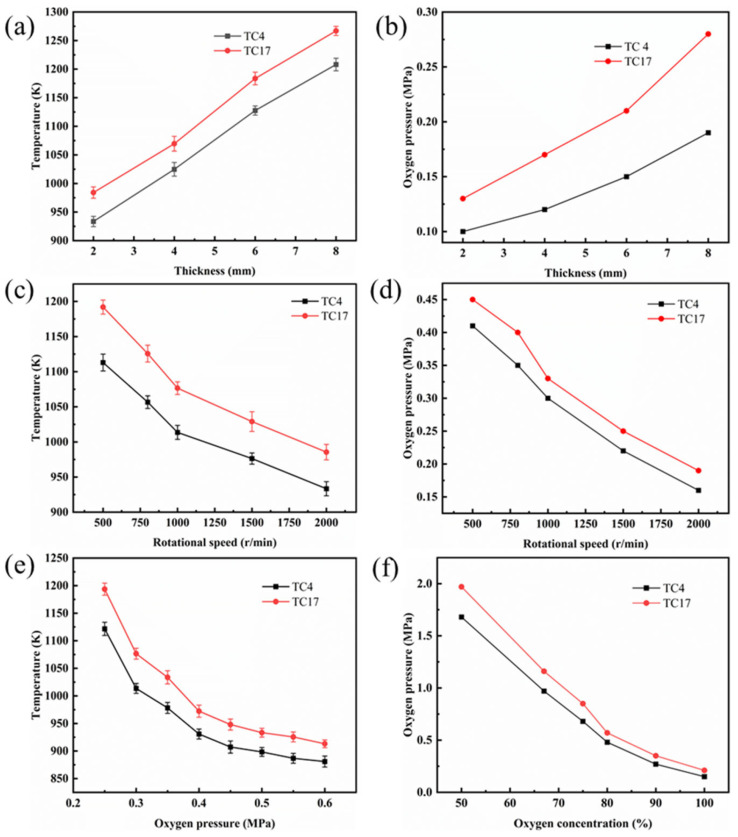
The combustion threshold temperatures and pressures of TC4 and TC17 alloys under the following conditions: (**a**,**b**) thickness; (**c**,**d**) rotational speed; (**e**) oxygen pressure; (**f**) oxygen concentration.

The critical ignition conditions of the TC4 and TC17 alloys under various experimental parameters are shown in Figure 10. This includes the critical ignition conditions for different sample thicknesses, rotational speeds, oxygen concentrations, and oxygen pressures. As illustrated in Figure 10a,b, when the experimental load is fixed at 200 N and the rotational speed is set at 1000 r/min, the critical combustion conditions are influenced by the sample size. As the thickness increases from 2 mm to 8 mm, the combustion threshold pressure of the TC4 alloy rises from 0.1 MPa to 0.19 MPa. Simultaneously, the combustion threshold temperature of the TC4 alloy increases from 933.5 K to 1208.1 K at 0.25 MPa oxygen pressure. The combustion threshold pressure of the TC17 alloy increases from 0.13 MPa to 0.28 MPa, while the combustion threshold temperature of the TC17 alloy rises from 984.1 K to 1276.9 K. As shown in Figure 10c,d, when the load is fixed at 200 N, the critical pressure and ignition temperature for the rubbing ignition decrease as the rotational speed increases. As the rotational speed increases from 500 r/min to 2000 r/min, the combustion threshold pressure of the TC4 alloy decreases from 0.41 MPa to 0.16 MPa. Simultaneously, the combustion threshold temperature of the TC4 alloy decreases from 1113 K to 932.4 K at 0.3 MPa oxygen pressure. The combustion threshold pressure of the TC17 alloy decreases from 0.46 MPa to 0.21 MPa as the rotational speed increases from 500 r/min to 2000 r/min. Concurrently, the combustion threshold temperature of the TC17 alloy drops from 1172 K to 985.5 K. The effects of oxygen pressure and oxygen concentration on ignition conditions were studied under a load of 200 N and a rotational speed of 1000 r/min. Figure 10e illustrates the combustion threshold temperatures of the TC4 and TC17 alloys under different oxygen pressures. The curve in the figure indicates that the ignition temperature of the titanium alloy decreases as the oxygen pressure increases. Specifically, as the oxygen pressure rises from 0.25 MPa to 0.60 MPa, the ignition temperature of the TC4 alloy decreases from 1121.6 K to 880.9 K, while the ignition temperature of the TC17 alloy drops from 1193.6 K to 913.1 K. Figure 10f depicts the critical pressure of the TC4 and TC17 alloys at different oxygen concentrations. The graph clearly demonstrates that the critical pressure of the titanium alloy decreases as the oxygen concentration increases. Specifically, when the oxygen concentration rises from 50% to 100%, the critical pressure of the TC4 alloy decreases from 1.68 MPa to 0.15 MPa, while the critical pressure of the TC17 alloy drops from 1.97 MPa to 0.21 MPa.

## 4. Discussion

### 4.1. Comparative Analysis of Ignition Process

According to the analysis of the micro-morphology and phase composition of the surfaces of the two alloys during the rubbing process, it can be inferred that the wear mechanism in the initial stage of rubbing (0.5 s) is primarily characterized by abrasive wear. At this time, the temperature of the rubbing surface remains relatively low, while the applied load generates both shear stress and normal stress on the micro-protrusions of the contact surface. Due to the repeated application of the load, the micro-protrusions on the surface are susceptible to plastic deformation and fracture when subjected to shear stress, leading to the formation of wear debris (see Figure 7a). After the onset of rubbing, the abrasive particles on the surface scratch the rubbing surface, increasing its roughness. Surface micro-scratches are a significant factor contributing to stress concentration, which adversely affects fatigue life and may result in “fatigue defects” during the rubbing process [30]. These micro-scratches can cause stress concentration, diminish the material’s fatigue resistance, lead to crack formation, and ultimately result in the material peeling off from the rubbing surface. As the rubbing process continues, the temperature of the rubbing surface rises, causing the alloy to soften. This increase in temperature results in a decrease in hardness and an increase in plastic deformation. Due to the different responses of metal materials to strain rates, as sliding friction persists the strain rate of the surface layer increases, leading to a dislocation slip rate that is lower than the deformation rate. This disruption in material continuity results in the formation of microcracks. When these microcracks propagate to the surface, fracture occurs (see Figure 7e), resulting in delamination wear. At the moment of ignition, significant spalling occurs on the friction surface, accompanied by obvious cracks, the formation of substantial amounts of oxides such as TiO and TiO_2_, and the presence of molten structures.

According to the comparative analysis of the ignition process of the two alloys during rubbing, it is evident that wear debris significantly influences the wear mechanism throughout the rubbing process. Prior to combustion in both alloys, extensive large-scale spalling takes place on the rubbing surface. A comparison of the microscopic morphology during the rubbing process reveals that, at 2.5 s, a protective friction layer forms on the rubbing surface of the TC17 alloy (see Figure 8e), whereas TC4 has already undergone significant material delamination (see Figure 7e). The reason may be that the TC4 alloy material has a high viscosity, which easily leads to adhesive forces on the wear surface, resulting in adhesive tearing when it is subjected to external forces [31]. Under conditions of friction, the ignition of titanium alloy occurs on the surface of fresh metal [32]. The TC4 alloy had already experienced significant material spalling at 2.5 s, resulting in island-like micro-protrusions or micro-fragments. Each micro-protrusion or micro-fragment has a primary metal surface that can undergo an initial reaction with oxygen, making it more susceptible to combustion under the same test conditions.

### 4.2. Thermodynamic Analysis of High-Speed Rubbing Ignition

The Frank-Kamenetskii model has been widely employed to investigate the characteristics of the self-heating ignition of substances [21]. Frank-Kamenetskii defined a dimensionless heat generation number, *δ*, which is also known as the Damkohler number, as shown in Equation (1):

(1) $$\delta =\frac{El^{2}}{\lambda RT^{2}}q\cdot k\frac{1}{1+\alpha {\left(1-C_{i}\right)}^{n}p^{n}}{\left(\frac{P}{p_{o}}\right)}^{n}\exp(-\frac{E}{RT})$$

where *k* is the pre-exponential factor, *E* is the activation energy, *n* is the reaction order, α is the adsorption coefficient, *R* is the molar gas constant, *q* is the heat of reaction per unit mass, *P* is the critical oxygen pressure, and *P*_0_ is the atmospheric pressure. *λ* represents the thermal conductivity, and *l* is the length of the sample. The critical condition of ignition is of the form *δ* = *δcr*. *δcr* is only related to the shape and size of the specimen and the energy input. The values of *δcr* can be found in the literature [33] and are shown in Equation (2):

(2) $$\delta_{cr}=N\left(\alpha \right)e^{-\frac{E}{HRT^{2}}\cdot \frac{2\pi r\omega \cdot \mu N\cdot \eta \cdot \alpha_{1}}{d\cdot b}}$$

*N*(*α*) is the critical value of *δ* without energy input, *N*(*α*) = 0.878 [34]; we see immediately from Equations (1) and (2) that

(3) $$0.878e^{-\frac{E}{HRT^{2}}\cdot \frac{2\pi r\omega \cdot \mu N\cdot \eta \cdot \alpha_{1}}{d\cdot b}}=\frac{El^{2}}{\lambda RT^{2}}kq\frac{1}{1+\alpha {\left(1-C_{i}\right)}^{n}p^{n}}{\left(\frac{P}{p_{o}}\right)}^{n}exp(\frac{-E}{RT})$$

where *µ* represents the coefficient of friction, *N* denotes the load, *ω* signifies the rotational speed, *r* stands for the radius of the rotor component, *η* is the thermal conversion coefficient, and *α*_1_ is the thermal distribution coefficient.

The reaction order, adsorption coefficient, and activation energy can be determined by fitting the model to the experimental data. This analysis includes the effects of diameter, oxygen concentration, rotational speed, load, and oxygen pressure on the critical ignition conditions.

#### Determination of the Reaction Order and Adsorption Coefficient

When *C_i_* is 0–1, the absorption coefficient needs to be confirmed. It is assumed that the change in *T* in the same sample under different oxygen concentrations can be ignored. According to Equation (3), the relationship between the oxygen concentration and the critical oxygen pressure can be obtained in a mixed atmosphere of oxygen and nitrogen, which satisfies the following equation:

(4) $${\left(C_{i}\frac{P}{p_{o}}\right)}^{n}=0.878exp\{\frac{E}{RT}-\frac{E}{RT^{2}\cdot H\cdot d\cdot b}\cdot 2\pi r\omega \cdot \mu N\cdot \eta \cdot \alpha_{1}\}\cdot \frac{\lambda RT^{2}}{El^{2}kq}\cdot \frac{1}{1+\alpha {\left(1-C_{i}\right)}^{n}p^{n}}$$

The above equation can be rewritten as follows:

(5) $${\left(C_{i}\frac{P}{p_{o}}\right)}^{n}=A+A\cdot \alpha {\left(1-C_{i}\right)}^{n}p^{n}$$

where $A=0.878exp\{\frac{E}{RT}-\frac{E}{RT^{2}\cdot H\cdot d\cdot b}\cdot 2\pi r\omega \cdot \mu N\cdot \eta \cdot \alpha_{1}\}\cdot \frac{\lambda RT^{2}}{El^{2}kq}$, which is regarded as constant.

1. The determination of the activation energy and pre-exponential factor

According to Equation (3), the relationship between the oxygen pressure and the critical ignition temperature for samples with the same thickness can be expressed as follows:

(6) $$lnp=-\frac{2\pi r\omega \cdot \mu N\cdot \eta \cdot \alpha_{1}}{n\cdot d\cdot b\cdot R\cdot H}\cdot \frac{1}{T^{2}}+\frac{2}{n}\mathrm{lnT}+\frac{1}{n}\frac{E}{R}\cdot \frac{1}{T}-\frac{1}{n}ln\left(\frac{El^{2}0.878}{\lambda R}kq\right)$$

The above equation can be rewritten as follows:

(7) $$lnp=\frac{B}{T^{2}}+C\mathrm{lnT}+\frac{D}{T}+F$$

where $B=-\frac{2\pi r\omega \cdot \mu N\cdot \eta \cdot \alpha_{1}}{n\cdot d\cdot b\cdot R\cdot H}$,$C=\frac{2}{n}$,$D=\frac{E}{nR}$,$F=\frac{1}{n}ln\left(\frac{El^{2}0.878}{\lambda R}kq\right)$, which is regarded as constant.

**Figure 11:**
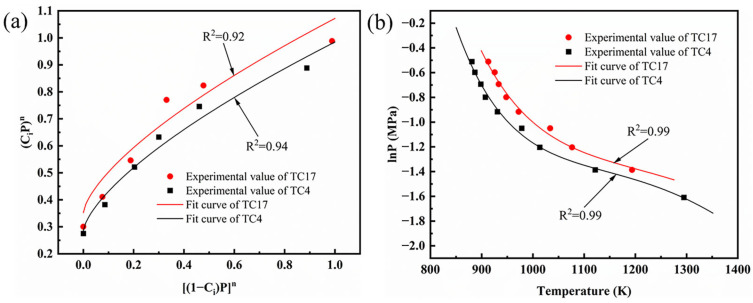
(**a**) the determination of adsorption coefficient (**b**) plots of T versus lnP.

To obtain the reaction order of the alloy with oxygen, the relationship between the oxygen concentration and the critical oxygen pressure was investigated (Figure 11a). According to Equation (4), the method of numerical fitting was applied to the curve of ${\left(C_{i}\frac{P}{p_{o}}\right)}^{n}$ and ${\left(1-C_{i}\right)}^{n}p^{n}$, represented by the curve in Figure 11a. The analysis determined that the values of α and n for the TC4 alloy were 2.45 and 0.68, respectively. In contrast, for the TC17 alloy, the values were 2.03 and 0.77. After substitution of the variables p and T from Figure 11b, the equation was solved using the least-squares method for B, C, D, and F: the values D, F, G, and M of the TC4 alloy under the rubbing condition were −5 × 10^7^, −78.89, 1.72 × 10^5^, and −669.93, respectively. This yielded the following values for the parameters of the activation energy and the pre-exponential factor for the titanium juvenile surfaces of the TC4 alloys: the activation energy was 97.18 kJ·mol^−1^, and the pre-exponential factor was 160.15 kg·m^−2^·s^−1^. Using the same method, the activation energy and pre-exponential factor of the TC17 alloy can be calculated, yielding values of 107.52 kJ·mol^−1^, and 147.4 kg·m^−2^·s^−1^, respectively.

According to the fitting results presented above, the key parameter values of the Frank-Kamenetskii model can be derived, as shown in Table 4. The reaction order is a parameter that describes the relationship between the rate of a chemical reaction and the concentration of each reactant. The higher reaction order of the TC17 alloy compared to the TC4 alloy indicates that concentration has a more significant impact on the reaction rate at the moment of ignition for the TC17 alloy. The adsorption coefficient quantifies a substance’s ability to adhere to a solid surface, which can reflect the reaction rate between metal and oxygen to some extent. The larger adsorption coefficient of the TC4 alloy compared to the TC17 alloy suggests that the reaction rate of the TC4 alloy at ignition is faster. Activation energy refers to the minimum energy required for a chemical reaction to occur at the moment of ignition, which can indicate the difficulty in ignition. The activation energy of the TC17 alloy is greater than that of the TC4 alloy, suggesting that the TC17 alloy exhibits better flame resistance than the TC4 alloy, which is consistent with the previous experimental results. The pre-exponential factor is a constant determined by the nature of the reaction and is independent of both the reaction temperature and the concentrations of the substances in the system.

The relationship between the ignition temperatures of the TC4 and TC17 alloys at different speeds under the same oxygen pressure can be derived from the logarithm of Equation (3), as follows:

(8) $$w=-\frac{d\cdot b\cdot R\cdot H}{2\pi r\cdot \mu N\cdot \eta \cdot \alpha_{1}\cdot E}\cdot ln\left(\frac{El^{2}0.878}{\lambda R}kqP^{n}\right)\cdot T^{2}-\frac{d\cdot b\cdot R\cdot H}{\pi r\cdot \mu N\cdot \eta \cdot \alpha_{1}\cdot E}\cdot T^{2}\cdot lnT-\frac{d\cdot b\cdot H}{2\pi r\cdot \mu N\cdot \eta \cdot \alpha_{1}}\cdot T$$

**Figure 12:**
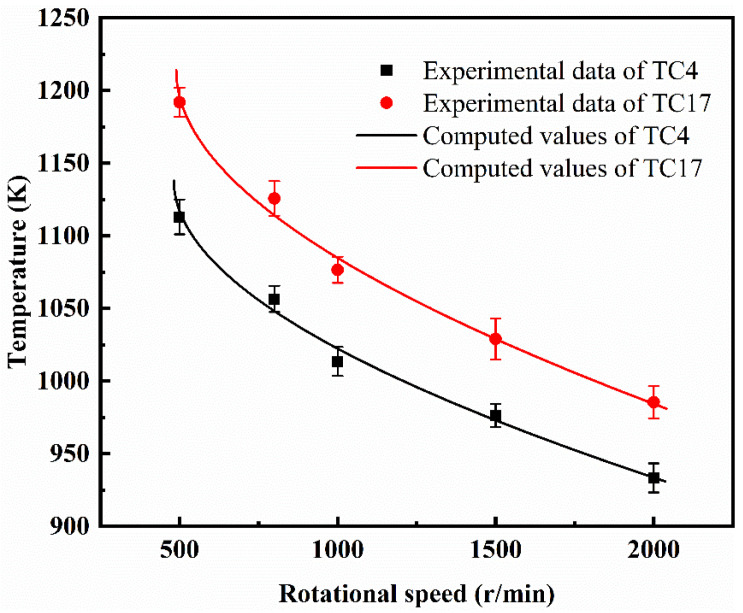
The experimental and computed values of ignition temperature at different rotational speeds of TC4 and TC17 alloys.

According to Equation (8), the ignition temperatures of the two alloys under an oxygen pressure of 0.3 MPa at different rotational speeds can be calculated, as shown in Figure 12. As can be seen in the graph, when the rotational speed is 800 r/min, the calculated value and the experimental value of the TC17 alloy exhibit the largest discrepancy, with a relative error of 2.06% at this point. Therefore, the Frank-Kamenetskii model can effectively predict the ignition temperature of titanium alloys under different rotational speeds and specific oxygen pressures. This indicates that the model is applicable to the ignition of titanium alloys under high-speed rubbing conditions.

## 5. Conclusions

This article compares the combustion behavior and critical ignition conditions of TC4 and TC17 alloys under friction-induced ignition conditions. The characteristic parameters of the Frank-Kamenetskii model under high-speed rubbing conditions have been determined. Additionally, the critical ignition temperature at different rotational speeds was predicted to validate the effectiveness of the model. This study offers theoretical support and establishes a foundation for further research on the ignition mechanisms and predictive models of titanium fires under the actual operating conditions of aero-engines. The following conclusions can be drawn:

(1) At the moment of ignition due to rubbing, both titanium alloys demonstrated a rapid increase in temperature, emitting a bright white light and generating intense heat. The critical pressure and ignition temperature for rub-induced ignition increased with larger sample sizes, while they decreased with higher rotational speeds, oxygen pressure, and oxygen concentration. Under the same test conditions, the critical pressure and ignition temperature for the rub-induced ignition of the TC17 alloy were both higher than those of the TC4 alloy.
(2) During the rubbing ignition process, the initial stage was primarily characterized by abrasive and adhesive wear. As the duration of rubbing increased, the material on the rubbing surface began to peel away, creating conditions conducive to ignition. At the moment of ignition, significant peeling occurred, along with visible cracks and molten structures, leading to the generation of a substantial amount of titanium oxide on the rubbing surface.
(3) The reaction order, absorption coefficient, and activation energy of the Frank-Kamenetskii model for the TC4 and TC17 alloys were determined. The ignition temperature of the TC4 and TC17 alloys at different speeds was predicted with a relative error of within 2.06%, indicating that the Frank-Kamenetskii model can be applied to describe the critical ignition conditions of bulk metals under frictional conditions.

## Figures and Tables

**Table 1:** The chemical compositions of TC4 and TC17 alloys (wt%).

Alloy	Ti	Al	Zr	Mo	Si	V	Sn	Cr
TC4	Bal.	6	-	-	-	4	-	-
TC17	Bal.	5	2	4	-	-	2	4

**Table 2:** The chemical compositions of TC4 and TC17 alloys (wt%).

Alloy	Room Temperature Mechanical Properties			High-Temperature Tensile Properties	
	Tensile Strength (Rm/MPa)	Elongation (%)	Reduction of Area (%)	Test Temperature(°C)	Tensile Strength (Rm/MPa)
TC4	825	10	25	400	620
TC17	1120	7	15	370	907

**Table 3:** The chemical compositions of rubbing surface during ignition moment (wt%).

Region	Ti	O	Al	Mo	Zr	V	Sn	Cr
1	50.88	43.16	3.77			2.19		
2	74.72	19.26	2.83			3.19		
3	63.77	28.76	4.54			2.53		
4	52.18	41.95	2.69	0.38	1.31		0.66	0.82
5	58.85	31.17	3.55	1.44	1.36		1.16	2.46
6	54.12	35.76	3.56	1.9	1.48		1.13	2.07

**Table 4:** The values of key parameters of the Frank-Kamenetskii model.

Alloy	n	α (MPa^−n^)	E (kJ/mol)	K (kg·m^−3^·s^−1^)
TC4	0.68	2.45	97.18	160.15
TC17	0.77	2.03	107.52	147.4

## Data Availability

The original contributions presented in this study are included in the article. Further inquiries can be directed to the corresponding author.

## Nomenclature

Nomenclature of parameters in the paper:

Standard symbol	Nomenclature	Dimension
*δ*	Frank-Kamenetskii parameter	-
*E*	activation energy	kJ·mol^−1^
*l*	sample length	m
*λ*	thermal conductivity	W·m^−1^ K^−1^
*R*	universal gas constant	J·mol^−1^·K^−1^
*T*	temperature	K
*q*	heat of reaction per unit mass	MJ·kg^−1^
*k*	pre-exponent	kg·m^−2^·s^−1^
*α*	adsorption coefficient	MPa^−n^
*C_i_*	oxygen concentration	-
*n*	reaction order	-
*P*	critical total pressure	MPa
*P*_0_	atmospheric pressure	MPa
*δcr*	Frank-Kameneskii parameters under critical conditions	-
*N*(*α*)	critical value of *δ* without energy input	-
*H*	gas heat transfer coefficient	W·m^−2^·K^−1^
*r*	rotor disk radius	m
*ω*	rotational speed	r/min
*μ*	frictional coefficient	
*N*	load	N
*η*	heat transfer coefficient	
*α*_1_	heat distribution coefficient	
*d*	sample thickness	m
*b*	sample width	m

## References

[1] Huang Z.H., Qu H.L., Deng C., Yang J.C. (2011). Development and application of titanium and titanium alloys for aviation. Mater. Rep..

[2] Yan L., Yu J., Zhong Y., Gu Y., Ma Y., Li W., Yan J., Ge Y., Yin J., Luo Y. (2020). Influence of scanning on nano crystalline β-Ti alloys fabricated by selective laser melting and their applications in biomedical science. J. Nanosci. Nanotechnol..

[3] Kosaka Y., Faller K., Fox S.P. (2004). Newly developed titanium alloy sheets for the exhaust systems of motorcycles and automobiles. JOM.

[4] Littman F.E., Church F.M., Kinderman E.M. (1961). A study of metal ignitions I. The spontaneous ignition of titanium. J. Less Common Met..

[5] Boyer R.R. (1996). An overview on the use of titanium in the aerospace industry. Mater. Sci. Eng. A.

[6] Shao L., Xie G., Liu X., Wu Y., Yu J., Hao Z., Lu W., Liu X. (2020). Combustion behaviour and mechanism of TC4 and TC11 alloys. Corros. Sci..

[7] Shao L., Xie G.L., Li H.Y., Lu W., Liu X., Yu J., Huang J. (2020). Combustion behavior and mechanism of Ti14 titanium alloy. Materials.

[8] Shao L., Li W., Li D., Xie G., Zhang C., Zhang C., Huang J. (2023). A review on combustion behavior and mechanism of Ti alloys for advanced aero-engine. J. Alloys Compd..

[9] Shao L., Wang Y., Xie G., Li H., Xiong J., Yu J., He G., Huang J. (2019). Combustion Mechanism of Alloying Elements Cr in Ti-Cr-V Alloys. Materials.

[10] Bolobov V.I. (2002). Mechanism of Self-Ignition of Titanium Alloys in Oxygen. Combust. Explos. Shock. Waves.

[11] Bolobov V.I. (2003). Possible Mechanism of Autoignition of Titanium Alloys in Oxygen. Combust. Explos. Shock. Waves.

[12] Bolobov V.I., Podlevskikh N.A. (2007). Mechanism of metal ignition due to fracture. Combust. Explos. Shock. Waves.

[13] Khaikin B.I., Bloshenko V.N., Merzhanov A.G. (1970). On the ignition of metal particles. Combust. Explos. Shock. Waves.

[14] Borisova Y.A., Sklyarov N.M. (1993). Fireproof titanium alloys. Phys. Metallogr..

[15] Liang X.Y., Mi G.B., Li P.J., Huang X., Cao C.X. (2020). Theoretical study on ignition of titanium alloy under high temperature friction condition. Acta Phys. Sin..

[16] Hernández-Acosta M.A., Martines-Arano H., Soto-Ruvalcaba L., Martínez-González C.L., Martínez-Gutiérrez H., Torres-Torres C. (2020). Fractional thermal transport and twisted light induced by an optical two-wave mixing in single-wall carbon nanotubes. Int. J. Therm. Sci..

[17] Mi G., Huang X., Cai J., Li S., Cao J., Cao C. (2016). Fireproof Property and Its Mechanism of A New High Temperature Titanium Alloy. Proceedings of the 13th World Conference on Titanium.

[18] Mi G.B., Cao C.X. (2014). Ignition Resistance Performance and Its Mechanism of TC17 Titanium Alloy for Aero-Engine. J. Aeronaut. Mater..

[19] Liang X., Mi G., Li P., Huang X., Cao C. (2021). Theoretical calculation of characteristics on titanium fire in aero-engine. J. Aeronaut. Mater..

[20] Gray B.F. (1975). Critical Behaviour in Chemically Reacting Systems III—An Analytical Criterion for Insensitivity. Combust. Flame.

[21] Thomas P.H. (1958). On the thermal conduction equation for self-heating materials with surface cooling. Trans. Faraday Soc..

[22] Gray B.F. (1969). Unified Theory of Explosions, Cool Flames and Two Stage Ignitions. Trans. Faraday Soc..

[23] Gray B.F. (2016). Spontaneous Combustion and Self-Heating. SFPE Handbook of Fire Protection Engineering.

[24] Garcia-Torrent J., Ramírez-Gómez A., Querol-Aragón E., Grima-Olmedo C., Medic-Pejic L. (2012). Determination of the risk of selfignition of coals and biomass materials. J. Hazard. Mater..

[25] He X., Restuccia F., Zhang Y., Hu Z., Huang X., Fang J., Rein G. (2020). Experimental Study of Self-heating Ignition of Lithium-Ion Batteries During Storage: Effect of the Number of Cells. Fire Technol..

[26] Wang C., Li Z., Dou C., Jiao Y., Li J., He G., Song X., Huang J., Zhang C. (2023). A Theoretical Description of the Ignition Conditions for TC17 Alloy in Oxygen-Enriched Atmospheres. Combust. Sci. Technol..

[27] (2018). Specification of Titanium and Titanium Alloy Bars and Forging Stocks for Aircraft.

[28] Wang C., Li J., Li Y., Dou C., Jin P., He G., Song X., Huang J., Zhang C. (2022). A Comparative Study on the Mathematic Models for the Ignition of Titanium Alloy in Oxygen-Enriched Environment. Metals.

[29] Straffelini G., Molinari A. (2011). Mild Sliding Wear of Fe–0.2%C, Ti–6%Al–4%V and Al-7072: A Comparative Study. Tribol. Lett..

[30] Zhang Q.Y., Ding H.Y., Zhou G.H., Wang S., Zhang L., Xia M., Guo X. (2019). Role of Fe_2_O_3_ in Dry Sliding Wear of a Titanium Alloy and Formation of Tribo-Layers. Rare Met. Mater. Eng..

[31] Jiang G., Zhao Z., Xiao G., Li S., Chen B., Zhuo X., Zhang J. (2022). Study of Surface Integrity of Titanium Alloy (TC4) by Belt Grinding to Achieve the Same Surface Roughness Range. Micromachines.

[32] Molinari A., Straffelini G., Tesi B., Bacci T. (1997). Dry sliding wear mechanisms of the Ti6Al4V alloy. Wear.

[33] Ouyang P., Mi G., Cao J., Huang X., He L., Li P. (2018). Microstructure Characteristics after combustion and fireproof mechanism of TiAl-based alloys. Mater. Today Commun..

[34] Gomez A., Wake G.C., Gray B.F. (1985). Friction and localized heat initiation of ignition: The asymmetrical slab and cylindrical annulus. Combust. Flame.

